# Supplementary material for: High coverage and equitable distribution of COVID-19 vaccine uptake in two vulnerable areas in Bangladesh
Source: PLOS Glob Public Health. 2025 Jan 17;5(1):e0004178. doi: 10.1371/journal.pgph.0004178 (PMC11741643; doi:10.1371/journal.pgph.0004178)
Supplement: S1 Text — (DOCX) [file pgph.0004178.s001.docx]

# TidiER-PH Narrative

## General Context

In Bangladesh, the COVID-19 pandemic started with the first cases diagnosed on March 8, 2020. Multiple waves successively hit the country over the following month forcing the country to rely on various strategies. During the first wave in March 2020, the national government initiated a strict lockdown and implemented it nationwide. Bangladesh was one of the first LMICs to launch a nationwide immunisation program against SARS-COv2 that started on February 7, 2021, and targeted at-risk populations. The program was extended to reach the entire population, and a massive vaccination campaign was set up. By November 13, 2022, almost 75% of the population had completed their vaccination schedule (two doses), while more than 86% had received at least one. However, many studies have shown that the vaccination coverage reached during COVID-19 reflects inequalities, especially since vulnerable populations might have a different level of access to vaccination than the general population. Yet, our findings from vulnerable settings in Bangladesh showed a vaccination coverage similar to the average national, stating that Bangladesh could be an exemplar story for COVID-19 vaccination. Here we aim to unfold the process that could explain how Bangladesh reached such a level of coverage and see how its lessons can be applied to similar situations.

## Method

We gathered data from two vulnerable communities, urban and rural: a slum in the capital Dhaka and a village in the southwest area of the country. We conducted 26 interviews from October to November 2022 that explored the perception of the covid-19 vaccination program. Interviewees were over 21, living in the area, and agreed to participate. All interviews were conducted in Bangla and translated later on. Champ to describe the COVID-19 vaccination program strategy (from February 2021 to September 2022) and explain the mass participation, we used the TIDieR-PHP checklist.

## Results

Why – For each stage of vaccination (1st dose, then 2nd doses), the purpose was to reach the same level of uptake coverage as the national level (above 80%) in rural areas or in slums which are the most vulnerable communities.

What Materials – Content of discourse provided by the community leader involved:

- Information about the importance of vaccination and its efficacy against Covid-19
- Information about the consequences of non-vaccination: work restriction, no access to health care centres, shops, administrations, etc.
- Information was delivered regarding the purpose of vaccination and the location of vaccination sites. No specific materials were provided because the intervention is based on information about vaccination delivered orally (public speaker and face-to-face).

What and How – For most respondents, vaccine hesitancy was due to the *“fear of being vaccinated”* as rumours were spread that those vaccinated would die from the prick. Their perceptions regarding vaccination shifted when the fear of dying from COVID surpassed their fear of vaccination. Some had strong resistances lifted solely by administrative restrictions: work restrictions, no access to health care centres, shops, administrations, etc. A micro-level community reinforcement for awareness was put in place in these communities. It relied on public speaker announcements of vaccination sites and at-home visits to overcome hesitancy. A two-step approach was taken by the community leaders involved in the project. First, home visits were performed randomly in the slum and on the outskirts of the rural area, where intervention providers met with the adults in the household to discuss. Secondly, they identified people hosting unvaccinated or partially vaccinated and returned to their households to explain the importance of vaccination after the first and the second round of immunisation. Leaders visited the households and discussed face-to-face with individuals. The discussions ranged from awareness discussions to threats according to their hesitancy level. As a community leader stated: “*We requested and forced the community people to take vaccination*”.

No other modes of delivery were provided. Intervention providers and the targeted population were from the same community.

Who provided – A context-related program relying on health workers, community leaders, and official representatives at the micro level.

Participants were:

- in the slum: local community leaders;
- in a rural area: chairman, policeman.

No specific training related to counselling and vaccination hesitancy and awareness was provided.

## Where – This intervention was carried out at the micro and household levels in each vulnerable community.

## When and how often – Intervention was delivered from February 7 to the first semester of 2022. The intervention delivery matched the vaccination phases for dose one and dose 2. It started alongside the availability of the vaccination's first dose.

Planned variation – The information content varied according to the leaders, the vaccination status, and the targeted population's vaccination hesitancy level.

How well – For each stage of vaccination (1st dose, then 2nd doses), leaders went to the selected households. When vaccine hesitancy persisted, intervention providers modified their discourse and added threats:

- Being listed and reported to the governmental authorities (slum and rural).
- They are being displaced (slum).
- No longer receiving food aid (slum).
