# Supplementary material for: High coverage and equitable distribution of COVID-19 vaccine uptake in two vulnerable areas in Bangladesh
Source: PLOS Glob Public Health. 2025 Jan 17;5(1):e0004178. doi: 10.1371/journal.pgph.0004178 (PMC11741643; doi:10.1371/journal.pgph.0004178)
Supplement: S1 Table — (DOCX) [file pgph.0004178.s005.docx]

S1 Table – Differences in explanatory variables by vaccine status for Duaripara

| Outcome variable | Taken at least two doses | | *p*-value | Taken two- and a booster-dose | | *p*-value |
| --- | --- | --- | --- | --- | --- | --- |
|  | Yes  (N = 996) | No  (N = 243) |  | Yes  (N = 173) | No  (N = 1066) |  |
| **Age** |  |  |  |  |  |  |
| 18-40 years | 680 (77.6%) | 196 (22.4%) | <0.001 | 96 (11.0%) | 780 (89.0%) | <0.001 |
| Above 40 | 316 (87.1%) | 47 (12.9%) |  | 77 (21.2%) | 286 (78.8%) |  |
| **Gender** |  |  |  |  |  |  |
| Male | 487 (83.1%) | 99 (16.9%) | 0.022 | 88 (15.0%) | 498 (85.0%) | 0.310 |
| Female | 509 (77.9%) | 144 (22.1%) |  | 85 (13.0%) | 568 (87.0%) |  |
| **Marital status** |  |  |  |  |  |  |
| Others | 106 (83.5%) | 21 (16.5%) | 0.357 | 18 (14.2%) | 109 (85.8%) | 0.942 |
| Currently married | 890 (80.0%) | 222 (20.0%) |  | 155 (13.9%) | 957 (86.1%) |  |
| **Education** |  |  |  |  |  |  |
| No education | 364 (84.3%) | 68 (15.7%) | 0.002 | 56 (13.0%) | 376 (87.0%) | 0.090 |
| Primary or less | 365 (81.3%) | 84 (18.7%) |  | 55 (12.3%) | 394 (87.7%) |  |
| Above primary | 267 (25.4%) | 91 (74.6%) |  | 62 (17.3%) | 296 (82.7%) |  |
| **Occupation** |  |  |  |  |  |  |
| Others | 281 (71.9%) | 110 (28.1%) | <0.001 | 50 (12.8%) | 341 (87.2%) | <0.001 |
| Business or self-employed | 204 (85.0%) | 36 (15.0%) |  | 37 (15.4%) | 203 (84.6%) |  |
| Service | 204 (84.6%) | 37 (15.4%) |  | 53 (22.0%) | 188 (78.0%) |  |
| Day labor | 307 (83.6%) | 60 (16.4%) |  | 33 (9.0%) | 334 (91.0%) |  |
| **Relationship with HH head** |  |  |  |  |  |  |
| Household-head | 493 (85.1%) | 86 (14.9%) | <0.001 | 82 (14.2%) | 497 (85.8%) | 0.829 |
| Spouse | 393 (77.2%) | 116 (22.8%) |  | 68 (13.4%) | 441 (86.6%) |  |
| Others | 110 (72.8%) | 41 (27.2%) |  | 23 (15.2%) | 128 (84.8%) |  |
| Household size [Mean (SD)] | 4.17 (1.56) | 4.24 (1.56) | 0.554 | 4.36 (1.86) | 4.16 (1.50) | 0.104 |
| Monthly income [Mean (SD)] | 18894 (10331) | 18096 (8984) | 0.269 | 21525 (12963) | 18285 (9464) | <0.001 |
| **Had access to television** |  |  |  |  |  |  |
| Yes | 506 (81.7%) | 113 (18.3%) | 0.229 | 103 (16.6%) | 516 (83.4%) | 0.007 |
| No | 490 (79.0%) | 130 (21.0%) |  | 70 (11.3%) | 550 (88.7%) |  |
| **Had access to smart-phone** |  |  |  |  |  |  |
| Yes | 537 (80.5%) | 130 (19.5%) | 0.907 | 111 (16.6%) | 556 (83.4%) | 0.003 |
| No | 459 (80.2%) | 113 (19.8%) |  | 62 (10.8%) | 510 (89.2%) |  |
| **Member of a micro-credit** |  |  |  |  |  |  |
| Yes | 249 (82.2%) | 54 (17.8%) | 0.383 | 38 (12.5%) | 265 (87.5%) | 0.408 |
| No | 747 (79.9%) | 189 (20.1%) |  | 135 (14.4%) | 800 (85.6%) |  |
| **Reported any chronic illness** |  |  |  |  |  |  |
| Yes | 242 (84.0%) | 46 (16.0%) | 0.076 | 50 (17.4%) | 238 (82.6%) | 0.058 |
| No | 754 (79.3%) | 197 (20.7%) |  | 123 (12.9%) | 828 (87.1%) |  |
| **Ever took COVID-19 test** |  |  |  |  |  |  |
| Yes | 104 (83.2%) | 21 (16.8%) | 0.404 | 28 (22.4%) | 97 (77.6%) | 0.004 |
| No | 892 (80.1%) | 222 (19.9%) |  | 145 (13.0%) | 969 (87.0%) |  |
| Mental wellbeing [Mean (SD)] | 44.71 (23.22) | 46.35 (22.40) | 0.318 | 47.35 (23.67) | 44.65 (22.95) | 0.153 |
| **Migrated in past 12 months** |  |  |  |  |  |  |
| Yes | 50 (76.9%) | 15 (23.1%) | 0.470 | 11 (16.9%) | 54 (83.1%) | 0.479 |
| No | 946 (80.6%) | 228 (19.4%) |  | 162 (13.8%) | 1012 (86.2%) |  |
