# Supplementary material for: High coverage and equitable distribution of COVID-19 vaccine uptake in two vulnerable areas in Bangladesh
Source: PLOS Glob Public Health. 2025 Jan 17;5(1):e0004178. doi: 10.1371/journal.pgph.0004178 (PMC11741643; doi:10.1371/journal.pgph.0004178)
Supplement: S2 Table — (DOCX) [file pgph.0004178.s006.docx]

S2 Table – Differences in explanatory variables by vaccine status for Tala

| Outcome variable | Taken at least two doses | | *p*-value | Taken two- and a booster-dose | | *p*-value |
| --- | --- | --- | --- | --- | --- | --- |
|  | Yes  (N = 1165) | No  (N = 98) |  | Yes  (N = 662) | No  (N = 601) |  |
| **Age** |  |  |  |  |  |  |
| 18-40 years | 602 (88.4%) | 79 (11.6%) | <0.001 | 299 (43.9%) | 382 (56.1%) | <0.001 |
| Above 40 | 563 (96.7%) | 19 (3.3%) |  | 363 (62.4%) | 219 (37.6%) |  |
| **Gender** |  |  |  |  |  |  |
| Male | 572 (93.3%) | 41 (6.7%) | 0.167 | 325 (53.0%) | 288 (47.0%) | 0.677 |
| Female | 593 (91.2%) | 57 (8.8%) |  | 337 (51.9%) | 313 (48.1%) |  |
| **Marital status** |  |  |  |  |  |  |
| Others | 102 (93.6%) | 7 (6.4%) | 0.585 | 59 (54.1%) | 50 (45.9%) | 0.708 |
| Currently married | 1063 (92.1%) | 91 (7.9%) |  | 603 (52.2%) | 551 (47.8%) |  |
| **Education** |  |  |  |  |  |  |
| No education | 226 (93.4%) | 16 (6.6%) | 0.051 | 135 (55.8%) | 107 (44.2%) | 0.361 |
| Primary or less | 360 (94.5%) | 21 (5.5%) |  | 203 (53.3%) | 178 (46.7%) |  |
| Above primary | 579 (90.5%) | 61 (9.5%) |  | 324 (50.6%) | 316 (49.4%) |  |
| **Occupation** |  |  |  |  |  |  |
| Others | 476 (89.1%) | 58 (10.9%) | 0.002 | 270 (50.6%) | 264 (49.4%) | 0.228 |
| Agriculture | 294 (95.8%) | 13 (4.2%) |  | 172 (56.0%) | 135 (44.0%) |  |
| Business or self-employed | 156 (95.7%) | 7 (4.3%) |  | 92 (56.4%) | 71 (43.6%) |  |
| Day labor | 239 (92.3%) | 20 (7.7%) |  | 128 (49.4%) | 131 (50.6%) |  |
| **Relationship with HH head** |  |  |  |  |  |  |
| Household-head | 529 (94.3%) | 32 (5.7%) | 0.037 | 307 (54.7%) | 254 (45.3%) | 0.124 |
| Spouse | 433 (91.2%) | 42 (8.8%) |  | 249 (52.4%) | 226 (47.6%) |  |
| Others | 203 (89.4%) | 24 (10.6%) |  | 106 (46.7%) | 121 (53.3%) |  |
| Household size [Mean (SD)] | 4.11 (1.39) | 4.17 (1.32) | 0.685 | 4.09 (1.41) | 4.15 (1.37) | 0.475 |
| Monthly income [Mean (SD)] | 14148.37 (10553.04) | 13289.39 (7685.55) | 0.431 | 14646.04 (12158.39) | 13460.13 (7881.49) | 0.042 |
| **Had access to television** |  |  |  |  |  |  |
| Yes | 535 (93.0%) | 40 (7.0%) | 0.330 | 335 (58.3%) | 240 (41.7%) | <0.001 |
| No | 630 (91.6%) | 58 (8.4%) |  | 327 (47.5%) | 361 (52.5%) |  |
| **Had access to smart-phone** |  |  |  |  |  |  |
| Yes | 551 (92.0%) | 48 (8.0%) | 0.749 | 294 (49.1%) | 305 (50.9%) | 0.024 |
| No | 614 (92.5%) | 50 (7.6%) |  | 368 (55.4%) | 296 (44.6%) |  |
| **Member of a micro-credit** |  |  |  |  |  |  |
| Yes | 505 (93.4%) | 36 (6.6%) | 0.204 | 295 (54.5%) | 246 (45.5%) | 0.193 |
| No | 660 (91.4%) | 62 (8.6%) |  | 367 (50.8%) | 355 (49.2%) |  |
| **Reported any chronic illness** |  |  |  |  |  |  |
| Yes | 384 (95.5%) | 18 (4.5%) | 0.003 | 232 (57.7%) | 170 (42.3%) | 0.010 |
| No | 781 (90.7%) | 80 (9.3%) |  | 430 (49.9%) | 431 (50.1%) |  |
| **Ever took COVID-19 test** |  |  |  |  |  |  |
| Yes | 48 (94.1%) | 3 (5.9%) | 0.609 | 31 (60.8%) | 20 (39.2%) | 0.222 |
| No | 1117 (92.2%) | 95 (7.8%) |  | 631 (52.1%) | 581 (47.9%) |  |
| Mental wellbeing [Mean (SD)] | 48.34 (20.14) | 49.96 (20.05) | 0.445 | 48.32 (20.56) | 48.63 (19.66) | 0.788 |
| **Migrated in past 12 months** |  |  |  |  |  |  |
| Yes | 110 (82.1%) | 24 (17.9%) | <0.001 | 51 (38.1%) | 83 (61.9%) | <0.001 |
| No | 1055 (93.4%) | 74 (6.6%) |  | 611 (54.1%) | 518 (45.9%) |  |
| Distance [in meter, Mean (SD)] | 831.92 (636.40) | 765.81 (527.46) | 0.318 | 806.24 (614.30) | 849.44 (643.92) | 0.223 |
