# Supplementary material for: High coverage and equitable distribution of COVID-19 vaccine uptake in two vulnerable areas in Bangladesh
Source: PLOS Glob Public Health. 2025 Jan 17;5(1):e0004178. doi: 10.1371/journal.pgph.0004178 (PMC11741643; doi:10.1371/journal.pgph.0004178)
Supplement: S3 Table — (DOCX) [file pgph.0004178.s007.docx]

S3 Table – Regression results presenting outcome variable as ordinal and logit-link function.

|  | Duaripara | Tala |
| --- | --- | --- |
| Outcome variable | No. of doses | No. of doses |
| Family distribution | Ordinal | Ordinal |
| Link function | Logit | Logit |
| Relative risk measure | OR | OR |
|  |  |  |
| **Age** |  |  |
| 18-40 years | **Reference** | **Reference** |
| Above 40 | 2.02*** | 2.62*** |
|  | (<0.01) | (<0.01) |
|  | 1.42 - 2.87 | 1.80 - 3.83 |
| **Gender** |  |  |
| Male | **Reference** | **Reference** |
| Female | 0.91 | 1.15 |
|  | (0.71) | (0.61) |
|  | 0.57 - 1.47 | 0.67 - 1.96 |
| **Marital status** |  |  |
| Others | **Reference** | **Reference** |
| Currently married | 0.69 | 0.65 |
|  | (0.20) | (0.17) |
|  | 0.39 - 1.22 | 0.35 - 1.20 |
| **Education** |  |  |
| No education | **Reference** | **Reference** |
| Primary or less | 1.05 | 0.90 |
|  | (0.78) | (0.67) |
|  | 0.75 - 1.48 | 0.56 - 1.45 |
| Above primary | 0.94 | 0.89 |
|  | (0.76) | (0.66) |
|  | 0.65 - 1.38 | 0.55 - 1.47 |
| **Occupation** |  |  |
| Others | **Reference** | **Reference** |
| Agriculture |  | 1.67** |
|  |  | (0.01) |
|  |  | 1.11 - 2.51 |
| Business or self-employed | 2.02*** | 1.94** |
|  | (<0.01) | (0.02) |
|  | 1.26 - 3.24 | 1.12 - 3.35 |
| Service | 3.14*** |  |
|  | (<0.01) |  |
|  | 2.03 - 4.86 |  |
| Day labor | 1.63** | 1.72** |
|  | (0.01) | (0.04) |
|  | 1.10 - 2.40 | 1.03 - 2.85 |
| **Relationship with HH head** |  |  |
| Household-head | **Reference** | **Reference** |
| Spouse | 1.12 | 1.02 |
|  | (0.66) | (0.93) |
|  | 0.68 - 1.85 | 0.58 - 1.81 |
| Others | 0.51** | 0.65 |
|  | (0.02) | (0.11) |
|  | 0.29 - 0.88 | 0.38 - 1.10 |
| Household size (standardized) | 0.97 | 1.06 |
|  | (0.76) | (0.61) |
|  | 0.82 - 1.15 | 0.84 - 1.34 |
| Monthly income (standardized) | 1.19* | 1.17 |
|  | (0.07) | (0.18) |
|  | 0.98 - 1.44 | 0.93 - 1.46 |
| =1 if had access to television | 1.38** | 1.69*** |
|  | (0.04) | (0.01) |
|  | 1.01 - 1.88 | 1.14 - 2.52 |
| =1 if had access to smartphone | 1.16 | 0.65** |
|  | (0.38) | (0.03) |
|  | 0.84 - 1.60 | 0.43 - 0.96 |
| =1 if member of a micro-credit | 0.98 | 1.44** |
|  | (0.88) | (0.03) |
|  | 0.70 - 1.36 | 1.03 - 2.01 |
| =1 if reported any chronic illness | 1.28 | 1.32 |
|  | (0.16) | (0.12) |
|  | 0.91 - 1.81 | 0.93 - 1.88 |
| =1 if ever took COVID-19 test | 1.23 | 1.00 |
|  | (0.38) | (0.99) |
|  | 0.77 - 1.95 | 0.46 - 2.20 |
| Mental wellbeing (standardized) | 1.06 | 1.12 |
|  | (0.45) | (0.25) |
|  | 0.91 - 1.22 | 0.93 - 1.35 |
| =1 if migrated in past 12 months | 1.10 | 0.32*** |
|  | (0.77) | (<0.01) |
|  | 0.59 - 2.04 | 0.20 - 0.53 |
| Distance from nearest healthcare facility (standardized) |  | 1.02 |
|  |  | (0.86) |
|  |  | 0.79 - 1.33 |
|  |  |  |
| Wald Chi-square statistic (*p*-value) | 76.1  (<0.001) | 90.8  (<0.001) |
| No. of villages | - | 42 |
| No. of households | 625 | 596 |
| No. of observations | 1239 | 1263 |

Notes: (a) For each dependent variable, coefficient, *P*-value (in parentheses), and 95% confidence interval are reported in three consecutive rows, (b) asterisks indicate statistical significance (*** p <0.01, ** p <0.05, * p <0.1).
