# Supplementary figures and images for: High coverage and equitable distribution of COVID-19 vaccine uptake in two vulnerable areas in Bangladesh
Source: PLOS Glob Public Health. 2025 Jan 17;5(1):e0004178. doi: 10.1371/journal.pgph.0004178 (PMC11741643; doi:10.1371/journal.pgph.0004178)

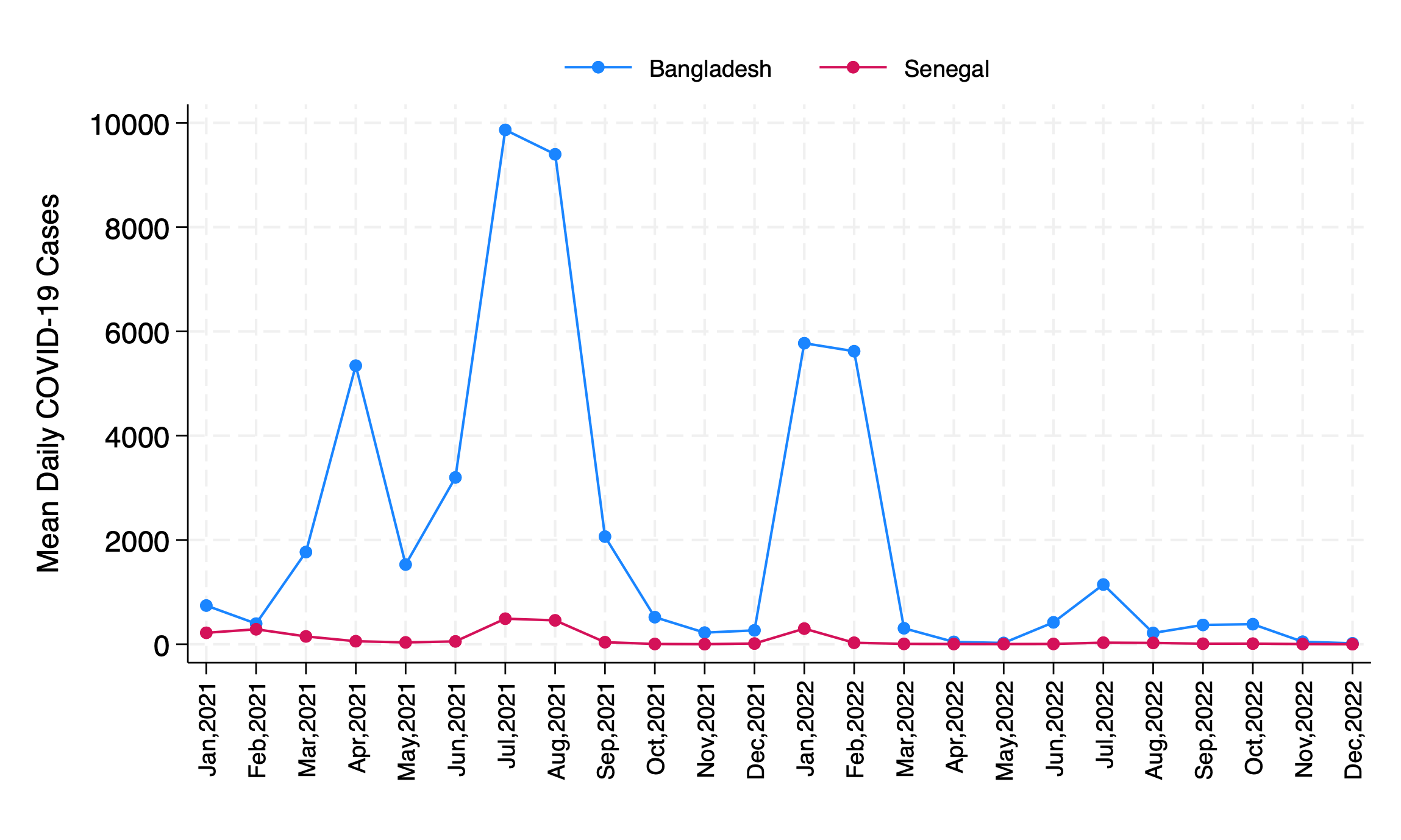

Supplement: S1 Fig — (TIFF) [file pgph.0004178.s008.tiff]

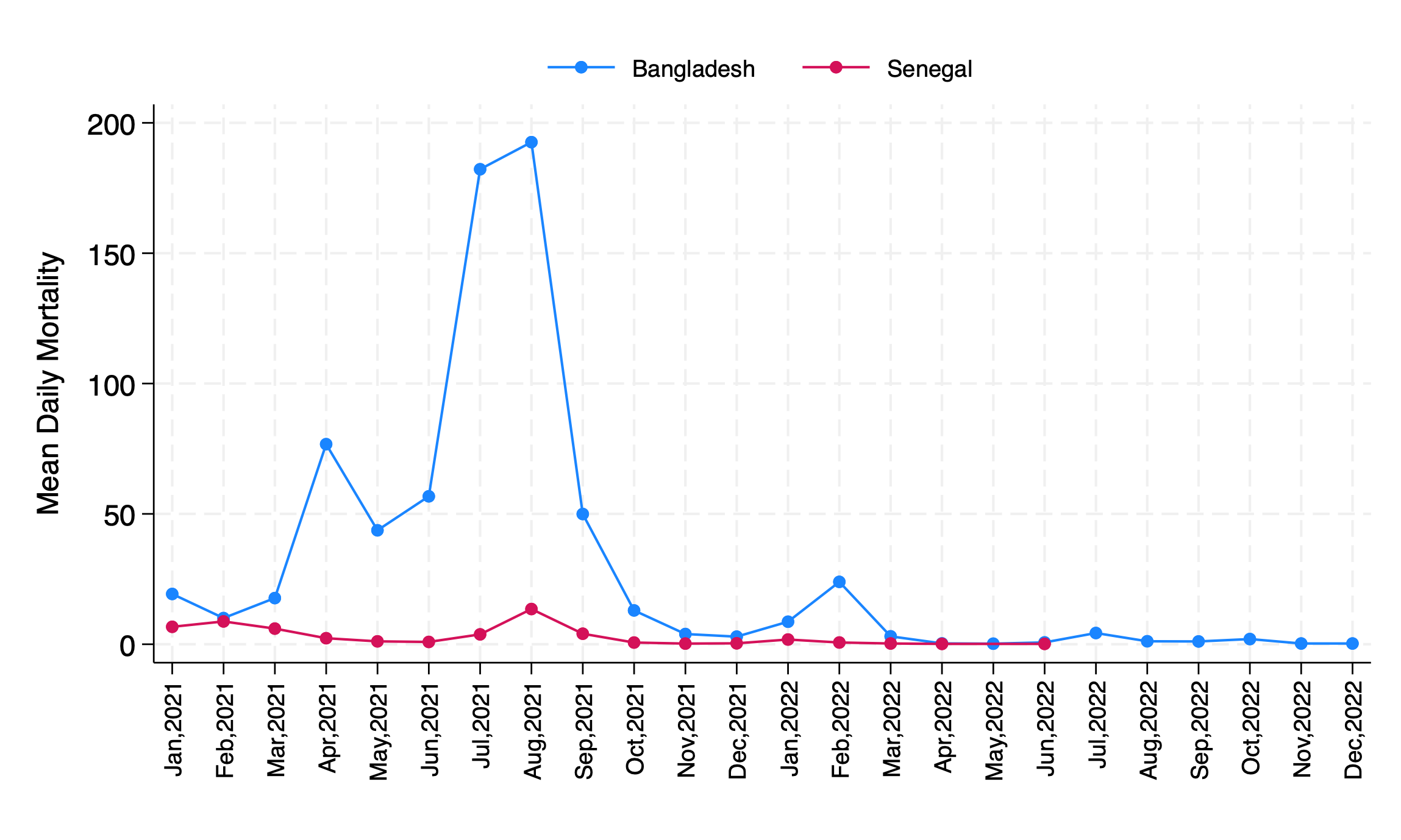

Supplement: S2 Fig — (TIFF) [file pgph.0004178.s009.tiff]
