# Supplementary material for: High coverage and equitable distribution of COVID-19 vaccine uptake in two vulnerable areas in Bangladesh
Source: PLOS Glob Public Health. 2025 Jan 17;5(1):e0004178. doi: 10.1371/journal.pgph.0004178 (PMC11741643; doi:10.1371/journal.pgph.0004178)
Supplement: Attachment — Submitted filename: PGPH-D-23-02525 (Review Report).pdf [file pgph.0004178.s010.pdf]

PGPH-D-23-02525

Title: High coverage and equitable distribution of COVID-19 vaccine uptake in two vulnerable areas in Bangladesh

This manuscript discusses the COVID-19 vaccine uptake and associated factors among adults in vulnerable areas in Bangladesh. The study conducted in Duaripara and Tala revealed high coverage rates for the first and second doses of the COVID-19 vaccine. Factors such as age, profession, access to television, membership with a micro-credit institution, smartphone access, and temporary migration status were associated with vaccine uptake in these areas. The study found that income was not associated with vaccine uptake, indicating equitable distribution. Geographical location of households did not correlate with vaccine uptake either. The success of high COVID-19 vaccine coverage in Bangladesh was attributed to the country's effective immunization programs, which focused on community mobilization and health education to generate demand. However, targeted interventions are suggested to ensure comprehensive coverage in vulnerable areas by addressing specific sociodemographic differences. While the results are interesting, I have some concerns as follows.

Q1: Regarding to the sample size determination in lines 145-158, the authors stated that

> "However, the people residing in slums are comparatively mobile. Hence, we estimated the sample size by adjusting for a 20% non-response rate."

In contrast, the authors adjusted the sample size for Tala by a design effect of 1.4 and a 10% non-response rate. I have three questions.

(a) Please explain how did the authors get idea of a design effect of 1.4. Please provide a reference for this.

(b) Please explain how did the authors determine 20% and 10% non-

response rate for Duaripara slum and Tala, respectively. Please provide some related references.

(c) According to table 2, and lines 312-313, the authors stated that

> “Only a tiny proportion, specifically 5.2% of the participants, reportedly migrated out of their Duaripara household within the last 12 months from the date of the survey.”

On the other hand, on lines 330-331, the authors stated that

> “About 10.6% of the participants in Tala reportedly migrated out of their households within the last 12 months.”

These results seem to tell me that the non-response rate in Duaripara is much lower than in Tala. These results contradict the authors’ assumptions of 20% and 10% non-response rate for Duaripara slum and Tala, respectively. Could the authors clarify this?

Q2: Table 1 presents variables, their measurement, and hypothesized direction of the coefficient. I have two questions.

(a) I'd like to know how the authors determine the direction of the coefficient, even if it is the "hypothetical" direction. We usually have some reference or legitimate reason. Please provide any explanations and add references you can find to support your hypothesis.

(b) Will the authors still get the correct results if the real direction is opposite to the "hypothetical" direction?

Q3: Please make sure your tables are displayed in a readable way. Please provide all definitions for any new notation. For example, in Table 4,

(1) what is the definition of “*”, “**”, “***”, and “****”?

(2) What is the meaning of the value “1.12 (0.04) 1.01-1.24” in row of “Above 40” and column of [1] in Table 4?

(3) More specifically, what is the notation “[1]”, “[2]”, and “[3]”, respectively, on Table 4. Are they “Model 1”, “Model 2” and “Model 3”, respectively? Or you

could just delete the notations “[1]”, “[2]”, and “[3]”. Please display your table more clearly and provide sufficient definitions for any new notations / symbols.

(4) If the “1.12 (0.04) 1.01-1.24” is “estimated value of IRR (p-value) 95%CI”, then why did the authors put “No. of villages”, “No. of households”and “No. of observations”in the same column in Table 4? Please make the table more readable.

(5) What is the “Base” means in Table 4? Do the authors mean that the group for Age 18-40 years is a “Reference” group and the group for Age “Above 40” are compared with the reference group? Then I recommend that the authors use “Reference” instead of “Base”, as the “Reference” group is more commonly used than the “Base” group.

	[1]	[2]	[3]	[4]	[5]	[6]
		Duaripara			Tala	
Outcome variable	No. of doses	Taken at least two doses	Taken at least two- and a booster-dose	No. of doses	Taken at least two doses	Taken at least two- and a booster-dose
Family distribution	Poisson	Bernoulli	Bernoulli	Poisson	Bernoulli	Bernoulli
Link function	Log	Logit	Logit	Log	Logit	Logit
Relative risk measure	IRR	OR	OR	IRR	OR	OR
**Age**						
18-40 years	**Base**	**Base**	**Base**	**Base**	**Base**	**Base**
Above 40	1.12**<br>(0.04)<br>1.01 - 1.24	1.51*<br>(0.09)<br>0.94 - 2.41	4.85***<br>(0.00)<br>2.43 - 9.68	1.14***<br>(0.00)<br>1.04 - 1.24	4.22***<br>(0.00)<br>1.93 - 9.21	2.78***<br>(0.00)<br>1.76 - 4.38
Gender						

……………….
……………….

	[1]	[2]	[3]	[4]	[5]	[6]
	[illegible]	[illegible]	[illegible]	[illegible]	[illegible]	[illegible]
Mental wellbeing (standardized)	1.01<br>(0.71)<br>0.97 - 1.05	0.99<br>(0.92)<br>0.82 - 1.19	1.22<br>(0.18)<br>0.91 - 1.62	1.01<br>(0.51)<br>0.97 - 1.06	1.17<br>(0.33)<br>0.85 - 1.61	1.09<br>(0.45)<br>0.87 - 1.37
=1 if migrated in past 12 months	0.98 — IRR<br>(0.86) — p-value<br>0.81 - 1.19 — 95% CI	0.94<br>(0.87)<br>0.43 - 2.03	1.63<br>(0.40)<br>0.53 - 5.00	0.87**<br>(0.04)<br>0.77 - 0.99	0.22***<br>(0.00)<br>0.10 - 0.49	0.35***<br>(0.00)<br>0.19 - 0.66
Distance from nearest healthcare facility (standardized)				1.00<br>(0.86)<br>0.96 - 1.03	1.19<br>(0.38)<br>0.81 - 1.76	0.94<br>(0.73)<br>0.69 - 1.30
Wald Chi-square statistic (*p*-value)	29.2<br>(0.063)	48.5<br>(<0.001)	45.7<br>(<0.001)	26.0<br>(0.166)	41.9<br>(0.003)	60.9<br>(<0.001)
No. of villages	-	-	-	42	42	42
No. of households	625	625	625	596	596	596
No. of observations	1239	1239	1239	1263	1263	1263

Q4: Please correct the typo.

access to a television, but negatively associated with access to a smartphones. Participants

Q5: In lines 410-413, the authors stated that

> "The results indicate a relatively egalitarian distribution of COVID-19 vaccination in these two vulnerable areas. The findings are quite different from the regular immunization programs for children in Bangladesh, where household wealth and the mother's education level significantly increase the likelihood of receiving full vaccination [29]."

Please explain the possible reasons in "Discussion" for the findings are quite different from the regular immunization programs for children in Bangladesh.

Q6: (Discussion): In lines 444-445, the authors stated that "For example, Bangladesh and Senegal are comparable in many aspects." In lines 456-459 of "Discussion", the authors stated that

> "An estimate in early 2021 revealed that the willingness to receive the COVID-19 vaccine was between 31% and 74.6% in Bangladesh [6,30,38]. People living in rural areas and slums (similar to our two sites) were more reluctant to be vaccinated [30]. However, the actual uptake rate surpassed the initial estimates and concerns."

I wonder how was the daily new case number of COVID-19 from early 2021 to the end of 2022 for these two countries. Please provide a figure to display (a) the daily new case number of COVID-19 from January 2021 to December 2022 and (b) COVID-19 vaccine coverage rate from January 2021 to December 2022. Please compare the figure in Bangladesh and in Senegal.

Please check whether the more severe the COVID pandemic, the more enthusiastic people are about getting vaccinated. Please add your findings in "Discussion".

Q7: Please explain in “Discussion” how to apply Bangladesh’s experience in increasing vaccine coverage to other countries, such as the United States, European countries, or other countries in Asia?”
