# Supplementary material for: High coverage and equitable distribution of COVID-19 vaccine uptake in two vulnerable areas in Bangladesh
Source: PLOS Glob Public Health. 2025 Jan 17;5(1):e0004178. doi: 10.1371/journal.pgph.0004178 (PMC11741643; doi:10.1371/journal.pgph.0004178)
Supplement: Attachment — Submitted filename: PGPH-D-23-02525.docx [file pgph.0004178.s011.docx]

1. The linear terms in the Poisson and logit models were inaccurately presented. Usually, the linear term in Poisson regression is linear only after log transformation, whereas the linear term in logit model is obtained after logit transformation. However, the linear term presented in both the models without making any transformation. As a result, the model formulation needs to be revised. Additionally, the author should verify how these changes impact the results obtained and report any changes accordingly.
2. “If we used – Number of doses of the COVID-19 vaccine – as the outcome variable, then we assumed the probability of taking a vaccine shot to follow a Poisson distribution and used a Log-link function.” Need to rethink the statement. Number of vaccine doses lie between 0 and 3 (maximum) which seems to be an ordinal form of variable. Under this assumption, some other models, i.e. ordinal logistic regression model with binomial link function, can help improve the results.
3. In model 1, the author employed multilevel generalized linear mixed effects models. However, it remains unclear which variable was treated as the random effect term. Additionally, considering urban slums like Duaripara, which typically fall under the jurisdiction of the Dhaka North City Corporation, it is important to clarify how the multilevel generalized linear mixed effects models were applied. Therefore, it is necessary to explicitly state the random effect term used in the analysis.
4. Don’t understand how the spatial analysis added extra value in the current context?
