# Supplementary material for: High coverage and equitable distribution of COVID-19 vaccine uptake in two vulnerable areas in Bangladesh
Source: PLOS Glob Public Health. 2025 Jan 17;5(1):e0004178. doi: 10.1371/journal.pgph.0004178 (PMC11741643; doi:10.1371/journal.pgph.0004178)
Supplement: Attachment — Submitted filename: Response to Reviewers_v2.docx [file pgph.0004178.s012.docx]

**PLOS Global Public Health**
**Ref:** PGPH-D-23-02525
**Title:** High coverage and equitable distribution of COVID-19 vaccine uptake in two vulnerable areas in Bangladesh.

**EDITOR’S COMMENTS**

1. *Please include a complete copy of PLOS’ questionnaire on inclusivity in global research in your revised manuscript. Our policy for research in this area aims to improve transparency in the reporting of research performed outside of researchers’ own country or community. The policy applies to researchers who have travelled to a different country to conduct research, research with Indigenous populations or their lands, and research on cultural artefacts. The questionnaire can also be requested at the journal’s discretion for any other submissions, even if these conditions are not met.  Please find more information on the policy and a link to download a blank copy of the questionnaire here:* [*https://journals.plos.org/globalpublichealth/s/best-practices-in-research-reporting*](https://journals.plos.org/globalpublichealth/s/best-practices-in-research-reporting)*. Please upload a completed version of your questionnaire as Supporting Information when you resubmit your manuscript.*

**Authors’ Reply:** Thank you for your suggestion. We have included a completed version of the inclusivity in global research in the supporting information. Following the journal requirements, we have also included a sub-section called ‘*Inclusivity in global research’* under the *Methods* secion. Please refer to page 16, lines 318-320 of the revised manuscript.

2. *Please amend your detailed Financial Disclosure statement. This is published with the article. It must therefore be completed in full sentences and contain the exact wording you wish to be published.*
(a) *State the initials, alongside each funding source, of each author to receive each grant.*
(b) *State what role the funders took in the study. If the funders had no role in your study, please state: “The funders had no role in study design, data collection and analysis, decision to publish, or preparation of the manuscript.”*
(c) *If any authors received a salary from any of your funders, please state which authors and which funders.*

*If you did not receive any funding for this study, please simply state: “The authors received no specific funding for this work.”*

**Authors’ Reply:** According to the suggestions, we have revised the funding information. Please refer to page 30, lines 739-742 of the revised manuscript.

3. *We do not publish any copyright or trademark symbols that usually accompany proprietary names, eg  ©, ®, ™  (e.g. next to drug or reagent names). Please remove all instances of trademark/copyright symbols throughout the text, including ® on page 15.*

**Authors’ Reply:** Thank you for pointing it out. We have removed the trademark from the revised manuscript. Please see page 15, line 301.

4. *We have noticed that you have uploaded Supporting Information files, but you have not included a list of legends. Please add a full list of legends for your Supporting Information files after the references list.*

**Authors’ Reply:** We have revised the manuscript by including a full list of legends for all the Supporting Information files. Please refer to page 34, lines 912-918 of the revised manuscript. I sincerely apologies for the inconveniences.

5. *Some material included in your submission may be copyrighted. According to PLOS’s copyright policy, authors who use figures or other material (e.g., graphics, clipart, maps) from another author or copyright holder must demonstrate or obtain permission to publish this material under the Creative Commons Attribution 4.0 International (CC BY 4.0) License used by PLOS journals. Please closely review the details of PLOS’s copyright requirements here: PLOS Licenses and Copyright. If you need to request permissions from a copyright holder, you may use PLOS's Copyright Content Permission form.*

*Please respond directly to this email or email the journal office and provide any known details concerning your material's license terms and permissions required for reuse, even if you have not yet obtained copyright permissions or are unsure of your material's copyright compatibility.*

*Potential Copyright Issues:*

*Figs 1 & 2: please (a) provide a direct link to the base layer of the map (i.e., the country or region border shape) and ensure this is also included in the figure legend; and (b) provide a link to the terms of use / license information for the base layer image or shapefile. We cannot publish proprietary or copyrighted maps (e.g. Google Maps, Mapquest) and the terms of use for your map base layer must be compatible with our CC-BY 4.0 license.*

*Note: if you created the map in a software program like R or ArcGIS, please locate and indicate the source of the basemap shapefile onto which data has been plotted.*

*If your map was obtained from a copyrighted source please amend the figure so that the base map used is from an openly available source. Alternatively, please provide explicit written permission from the copyright holder granting you the right to publish the material under our CC-BY 4.0 license.*

*Please note that the following CC BY licenses are compatible with PLOS license: CC BY 4.0, CC BY 2.0 and CC BY 3.0, meanwhile such licenses as CC BY-ND 3.0 and others are not compatible due to additional restrictions.*

**Authors’ Reply:** Thank you for pointing out this concern. We used QGIS to create the three maps included in this manuscript. The shapefiles used in this process are publicly available. We have shared the source(s) of the shapefiles in the reference list. However, the GPS coordinates of respondents’ households that we collected during the survey, were not shared complying with the de-identification policies. We understand and comply with the CC-BY 4.0 license requirements. We have also mentioned all this information and cited the source(s) of the shapefiles as notes beneath each of the figures. Please refer to the figures attached with the revised manuscript.

**REVIEWERS’ COMMENTS**

- *Have the authors made all data underlying the findings in their manuscript fully available (please refer to the Data Availability Statement at the start of the manuscript PDF file)?*

*The* [*PLOS Data policy*](https://journals.plos.org/globalpublichealth/s/data-availability) *requires authors to make all data underlying the findings described in their manuscript fully available without restriction, with rare exception. The data should be provided as part of the manuscript or its supporting information or deposited to a public repository. For example, in addition to summary statistics, the data points behind means, medians and variance measures should be available. If there are restrictions on publicly sharing data—e.g. participant privacy or use of data from a third party—those must be specified.*

*Reviewer #1: No*

*Reviewer #2: No*

**Authors’ Reply:** We have uploaded all the relevant data. Previously, data were submitted as a DTA file (i.e., compatible to Stata 17.0). Now, we have converted them to an Excel file and shared along with the revised manuscript. Additionally, we have revised the Data Availability Statement. Please see page 30, lines 735-736.

**COMMENTS FROM REVIEWER #2**

1. *The linear terms in the Poisson and logit models were inaccurately presented. Usually, the linear term in Poisson regression is linear only after log transformation, whereas the linear term in logit model is obtained after logit transformation. However, the linear term presented in both the models without making any transformation. As a result, the model formulation needs to be revised. Additionally, the author should verify how these changes impact the results obtained and report any changes accordingly.*

**Authors’ Reply:** Thank you very much for identifying the inaccuracies. We appreciate your valuable feedback. We have made necessary changes in all the equations. Please refer to page 13, equation 1; page 14, equations 2-6. However, the results remain the same, since only the equations were inaccurately presented. The software package used to fit the models accurately conducted all the necessary transformations. Hence, there was no impact on the findings reported in the manuscript.

2. *“If we used – Number of doses of the COVID-19 vaccine – as the outcome variable, then we assumed the probability of taking a vaccine shot to follow a Poisson distribution and used a Log-link function.” Need to rethink the statement. Number of vaccine doses lie between 0 and 3 (maximum) which seems to be an ordinal form of variable. Under this assumption, some other models, i.e. ordinal logistic regression model with binomial link function, can help improve the results.*

**Authors’ Reply:** Thank you. We really appreciate your valuable feedback. Following your suggestions, we have fitted multilevel generalized linear mixed-effects models considering the outcome variable as an ordinal variable and used a logit link function to estimate the coefficients. Our primary results remained qualitatively very similar, indicating robustness to the different estimation techniques applied. We have reported this in the *Results* section. Please refer to page 24, lines 613-616. We also reported the models in table S3 in the Supporting Information.

3. *In model 1, the author employed multilevel generalized linear mixed effects models. However, it remains unclear which variable was treated as the random effect term. Additionally, considering urban slums like Duaripara, which typically fall under the jurisdiction of the Dhaka North City Corporation, it is important to clarify how the multilevel generalized linear mixed effects models were applied. Therefore, it is necessary to explicitly state the random effect term used in the analysis.*

**Authors’ Reply:** We revised the manuscript mentioning that the data structure, for model 1, 2 and 3, were hierarchical representing two levels – individuals were nested in households. Please refer to page 13, lines 251-252. We also highlighted the lines where we explicitly state the random effect terms. Please refer to page 13, lines 256-257, and page 14, lines 276-277.

4. *Don’t understand how the spatial analysis added extra value in the current context?*

**Authors’ Reply:** The spatial analysis was conducted to examine whether there was any correlation between respondents' vaccination status and their place of residence. Tala is an area exposed to various climatic events, and not all villages within Tala are equally accessible or share the same resource settings. Thus, we hypothesized that the distribution of vaccines may have encountered different challenges across different parts of Tala. The spatial analysis also allowed us to visualize the distribution of COVID-19 vaccinations and assess whether they were equitably distributed within these two vulnerable areas.

**COMMENTS FROM REVIEWER #1**

1. *Regarding to the sample size determination in lines 145-158, the authors stated that: “However, the people residing in slums are comparatively mobile. Hence, we estimated the sample size by adjusting for a 20% non-response rate.” In contrast, the authors adjusted the sample size for Tala by a design effect of 1.4 and a 10% non-response rate. I have three questions:*
(a) *Please explain how did the authors get idea of a design effect of 1.4. Please provide a reference for this.*
(b) *Please explain how did the authors determine 20% and 10% non-response rate for Duaripara slum and Tala, respectively. Please provide some related references.*
(c) *According to table 2, and lines 312-313, the authors stated that – “Only a tiny proportion, specifically 5.2% of the participants, reportedly migrated out of their Duaripara household within the last 12 months from the date of the survey.” On the other hand, on lines 330-331, the authors stated that – “About 10.6% of the participants in Tala reportedly migrated out of their households within the last 12 months.” These results seem to tell me that the non-response rate in Duaripara is much lower than in Tala. These results contradict the authors’ assumptions of 20% and 10% non-response rate for Duaripara slum and Tala, respectively. Could the authors clarify this?*

**Authors’ Reply:** Thank you for your valuable feedback regarding our sample size estimations. Before addressing your specific queries, we would like to provide some context. This study is part of an exploratory research initiative aimed at understanding formal healthcare access among populations residing in climate-vulnerable areas. As we were unable to locate any existing literature that reported or estimated formal healthcare access in Duaripara and Tala, we conducted pilot studies in these locations. The data from these pilot studies were used to inform our sample size calculations. We have provided this information in the manuscript (please refer to pages 7 and 8, lines 145-155). However, given the potential variability in pilot estimates, we skeptically adjusted our calculations to reflect larger sample sizes to ensure robustness.

(a) Analyzing the data from the pilot study, we estimated to find an intra-cluster correlation coefficient of 0.01. We pre-decided to select 10 clusters in Tala. The initial sample size for Tala was 420; hence average cluster size was 42. Applying all these stats resulted in a design effect of 1.41.
(b) During the pilot, we experienced a non-response rate of around 10% in both Tala and Duaripara. However, we were skeptic about Duaripara since in contains temporary settlements and people were mostly residing in rented houses. We doubled the estimated non-response rate to adjust for potential variabilities. However, we finally managed to interview 625 households in Duaripara (please refer to page 8, lines 166-167). Therefore, the acutal non-response rate for Duaripara was around 9.9% and 9.4% for Tala.
(c) The definition of migration in our study is whether the respondents stayed outside of their home for 30 days or more within the last 12 months (please refer to Table 1 in page 10). Whereas non-response could result from events many events; for example, potential respondent may not provide consent to conduct the interview, we might not find the respondent while visiting the household, etc.
2. *Table 1 presents variables, their measurement, and hypothesized direction of the coefficient. I have two questions:*
(a) *I'd like to know how the authors determine the direction of the coefficient, even if it is the "hypothetical" direction. We usually have some reference or legitimate reason. Please provide any explanations and add references you can find to support your hypothesis.*
(b) *Will the authors still get the correct results if the real direction is opposite to the "hypothetical" direction?*

**Authors’ Reply:** Thank you for your feedback. We explained the reasons behind hypothesizing direction of the coefficients for age, relationship with household head, wealth and access to television, smartphone and microcredit institutions, and mental health in pages 11-12, lines 199-204, lines 210-213, lines 221-222 respectively. However, we revised the manuscript explaining the reason behind hypothesizing a negative association between chronic illness and vaccine uptake in page 11, lines 216-218.

3. *Please make sure your tables are displayed in a readable way. Please provide all definitions for any new notation. For example, in Table 4,*
(a) *what is the definition of “*”, “**”, “***”, and “****”?*
(b) *What is the meaning of the value “1.12 (0.04) 1.01-1.24” in row of “Above 40” and column of [1] in Table 4?*
(c) *More specifically, what is the notation “[1]”, “[2]”, and “[3]”, respectively, on Table 4. Are they “Model 1”, “Model 2” and “Model 3”, respectively? Or you could just delete the notations “[1]”, “[2]”, and “[3]”. Please display your table more clearly and provide sufficient definitions for any new notations / symbols.*
(d) *If the “1.12 (0.04) 1.01-1.24” is “estimated value of IRR (p-value) 95%CI”, then why did the authors put “No. of villages”, “No. of households”and “No. of observations”in the same column in Table 4? Please make the table more readable.*
(e) *What is the “Base” means in Table 4? Do the authors mean that the group for Age 18-40 years is a “Reference” group and the group for Age “Above 40” are compared with the reference group? Then I recommend that the authors use “Reference” instead of “Base”, as the “Reference” group is more commonly used than the “Base” group.*

**Authors’ Reply:** Thank you for your valuable feedback. We have revised Table 4 according to your suggestions. We have included notes to guide readers understand the results, removed terms that could cause confusions. Please refer to page 21 and 22 to see the changes.

4. Please correct the typo – “but negatively associated with access to a smartphones”.

**Authors’ Reply:** Thank you for bringing this to our attention. We have revised the manuscript, correcting the typo you identified as well as addressing other similar typographical errors.

5. *In lines 410-413, the authors stated that – “The results indicate a relatively egalitarian distribution of COVID-19 vaccination in these two vulnerable areas. The findings are quite different from the regular immunization programs for children in Bangladesh, where household wealth and the mother’s education level significantly increase the likelihood of receiving full vaccination [29].” Please explain the possible reasons in “Discussion” for the findings are quite different from the regular immunization programs for children in Bangladesh.*

**Authors’ Reply:** This is a very important programmatic aspect of the results of this study. Unfortunately, we do not have the precise data to understand these results, but in this section, we propose a few lines of thought and suggest the need for a qualitative study, see page 25-26.

6. *(Discussion): In lines 444-445, the authors stated that “For example, Bangladesh and Senegal are comparable in many aspects.” In lines 456-459 of “Discussion”, the authors stated that – “An estimate in early 2021 revealed that the willingness to receive the COVID-19 vaccine was between 31% and 74.6% in Bangladesh [6,30,38]. People living in rural areas and slums (similar to our two sites) were more reluctant to be vaccinated [30]. However, the actual uptake rate surpassed the initial estimates and concerns.” I wonder how was the daily new case number of COVID-19 from early 2021 to the end of 2022 for these two countries. Please provide a figure to display (a) the daily new case number of COVID-19 from January 2021 to December 2022 and (b) COVID-19 vaccine coverage rate from January 2021 to December 2022. Please compare the figure in Bangladesh and in Senegal.*

*Please check whether the more severe the COVID pandemic, the more enthusiastic people are about getting vaccinated. Please add your findings in “Discussion”.*

**Authors’ Reply:** We appreciate your valuable feedback. Following your suggestion, we have consulted the WHO’s COVID-19 dashboard data and plotted graphs of average daily new cases identified, and daily deaths occurred from January 2021 to December 2022 in Bangladesh and Senegal. The figures were shared as Supporting Information (Please refer to figure S1 and S2). Compared to Senegal, the daily new COVID-19 cases and mortalities were considerably higher in Bangladesh. Based on the findings, we revised the manuscript mentioning that the discrepancy between initial willingness to vaccinate and actual vaccination rate could also be attributed to the more severe COVID-19 outbreak experienced in Bangladesh compared to Senegal. Please refer to pages 27-28, lines 588-593.

7. *Please explain in “Discussion” how to apply Bangladesh’s experience in increasing vaccine coverage to other countries, such as the United States, European countries, or other countries in Asia?”*

**Authors’ Reply:** Thank you for your comment, but it seems to us that in the last sections of the Discussion, the favorable elements have been highlighted, such as community participation, good relations between the state and civil society and local involvement. However, we have added a sentence to remind that these countries can learn from the Bangladesh experience, see page 29.
